# Akirin2 is modulated by miR-490-3p and facilitates angiogenesis in cholangiocarcinoma through the IL-6/STAT3/VEGFA signaling pathway

**DOI:** 10.1038/s41419-019-1506-4

**Published:** 2019-03-18

**Authors:** Kaiming Leng, Yi Xu, Pengcheng Kang, Wei Qin, Hailong Cai, Hao Wang, Daolin Ji, Xingming Jiang, Jinglin Li, Zhenglong Li, Lining Huang, Xiangyu Zhong, Xueying Sun, Zhidong Wang, Yunfu Cui

**Affiliations:** 1 Department of Hepatopancreatobiliary Surgery, The Second Affiliated Hospital of Harbin Medical University, 150081 Harbin, China; 2 The Key Laboratory of Myocardial Ischemia, Harbin Medical University, Ministry of Education, Heilongjiang, China; 3 Department of Anesthesiology, The Fourth Affiliated Hospital of Harbin Medical University, 150081 Harbin, China; 4 Key Laboratory of Hepatosplenic Surgery, Department of General Surgery, The First Affiliated Hospital of Harbin Medical University, Harbin, 150001 China

## Abstract

Akirin2 is a key regulator of embryonic development and the innate immunity response. However, this regulator’s role in tumorigenesis especially in cholangiocarcinoma (CCA) development has not been thoroughly elucidated to date. In the current work, we used RT-qPCR, western blot analysis, and immunohistochemistry (IHC) to explore the expression level of Akirin2, and the relationship between Akirin2 levels and clinicopathological characteristics was evaluated. The biological functions of Akirin2 were examined in vitro and in vivo by using a lentiviral vector system. Luciferase reporter assays were applied to detect the direct binding relationship between the 3′-UTR of Akirin2 mRNA and miR-490-3p. The results showed that Akirin2 was overexpressed in CCA and this upregulation was associated with a shorter overall survival. Silencing or overexpressing Akirin2 by lentiviral approaches significantly influenced CCA cell proliferation, migration, invasion, and angiogenesis. An in vivo tumor model further validated the oncogenic effect of Akirin2 on CCA cell growth, metastasis, and angiogenesis. Mechanistic studies demonstrated that Akirin2 induced angiogenesis by increasing the expression of VEGFA by activating the IL-6/STAT3 signaling pathway. Akirin2 promoted cell migratory and invasive potential by affecting the epithelial–mesenchymal transition (EMT) process. In addition, Akirin2 expression was negatively controlled by miR-490-3p in CCA cells, and miR-490-3p attenuated cell migration and angiogenesis in CCA cells by silencing Akirin2. Taken together, the data indicated that Akirin2 could be regulated by miR-490-3p at the posttranscriptional level and facilitate CCA cell progression via the IL-6/STAT3/VEGFA signaling pathway. The present study may expedite the development of novel therapeutic strategies for CCA.

## Background

Cholangiocarcinoma (CCA) represents a diverse group of highly aggressive epithelial cancers originating from malignant transformation of cholangiocytes throughout the entire biliary tree^1^. The overall incidence and mortality rates of CCA, especially intrahepatic CCA, have increased considerably worldwide over the past four decades^2–4^. Unfortunately, most CCA patients are detected in advanced stages, losing the chance for curative surgical resection. The current first-line chemotherapy regimen (cisplatin plus gemcitabine) is of limited effectiveness, leaving patients with a median overall survival (OS) of <1 year after diagnosis^5^. Therefore, improving our understanding of tumor biology and the molecular pathogenesis of CCA is essential to develop personalized medicine and targeted therapies.

Genetically, the pathogenesis of CCA is complex and involved in dysregulation of numerous oncogenic drivers and tumor suppressors such as VEGF, BRAF, TP53, KRAS, SMAD4, IDH1/2, FGFR, BAP1, and MCL1^6–12^. There is an urgent need to demonstrate the underlying molecular mechanisms regulating CCA tumor growth, metastasis, and angiogenesis to establish effective anti-CCA therapeutic strategies.

Akirins have been identified as a group of highly evolutionary conserved nuclear factors. At least two Akirin family members, named Akirin1 and Akirin2, are present in humans and mice. Akirin2 is a key regulator of embryonic development in mice and when Akirin2 is deleted, no embryos are recovered as early as embryonic day 9.5^13^. Akirin2 is also required for the innate immunity response and the nuclear factor-kappa B (NF-κB)  signaling pathway that lead to the production of IL-6 in mice^13^. In addition, it has been reported that Akirin2 is critical for limb formation in mice^14^, and is essential for a wide variety of roles during neuronal development in Xenopus and mice^15,16^. Knockout of Akirin2 leads to soft-tissue syndactyly and neural apoptosis in mice. Akirin2 dysregulation has also been shown in several rat tumor cell lines^17–19^. Akirin2 is upregulated in human primary glioblastomas, and confers chemoresistance to glioblastomas and imatinib resistance to chronic myeloid leukemia^20,21^. However, whether Akirin2 promotes angiogenesis, or has other functions in CCA warrants further investigation.

In this work, we first documented that Akirin2 was significantly upregulated in human CCA through a mechanism by which miR-490-3p releases its inhibition of Akirin2 mRNA. The overexpression of Akirin2 was closely related to unfavorable prognosis in the patients with CCA. In addition, Akirin2 was identified as an oncogene that could promote CCA cell proliferation, metastasis, and angiogenesis both in vitro and in vivo. Furthermore, our data revealed that Akirin2 induced angiogenesis by increasing the expression of vascular endothelial growth factor A (VEGF) through activating the interleukin-6/signal transducers and activators of transcription 3 (IL-6/STAT3) signaling pathway. These findings indicate that Akirin2 may be regarded as a new effective therapeutic target for CCA.

## Results

### Akirin2 is upregulated in human CCAs and predicts a poor outcomes

**Fig. 1:**
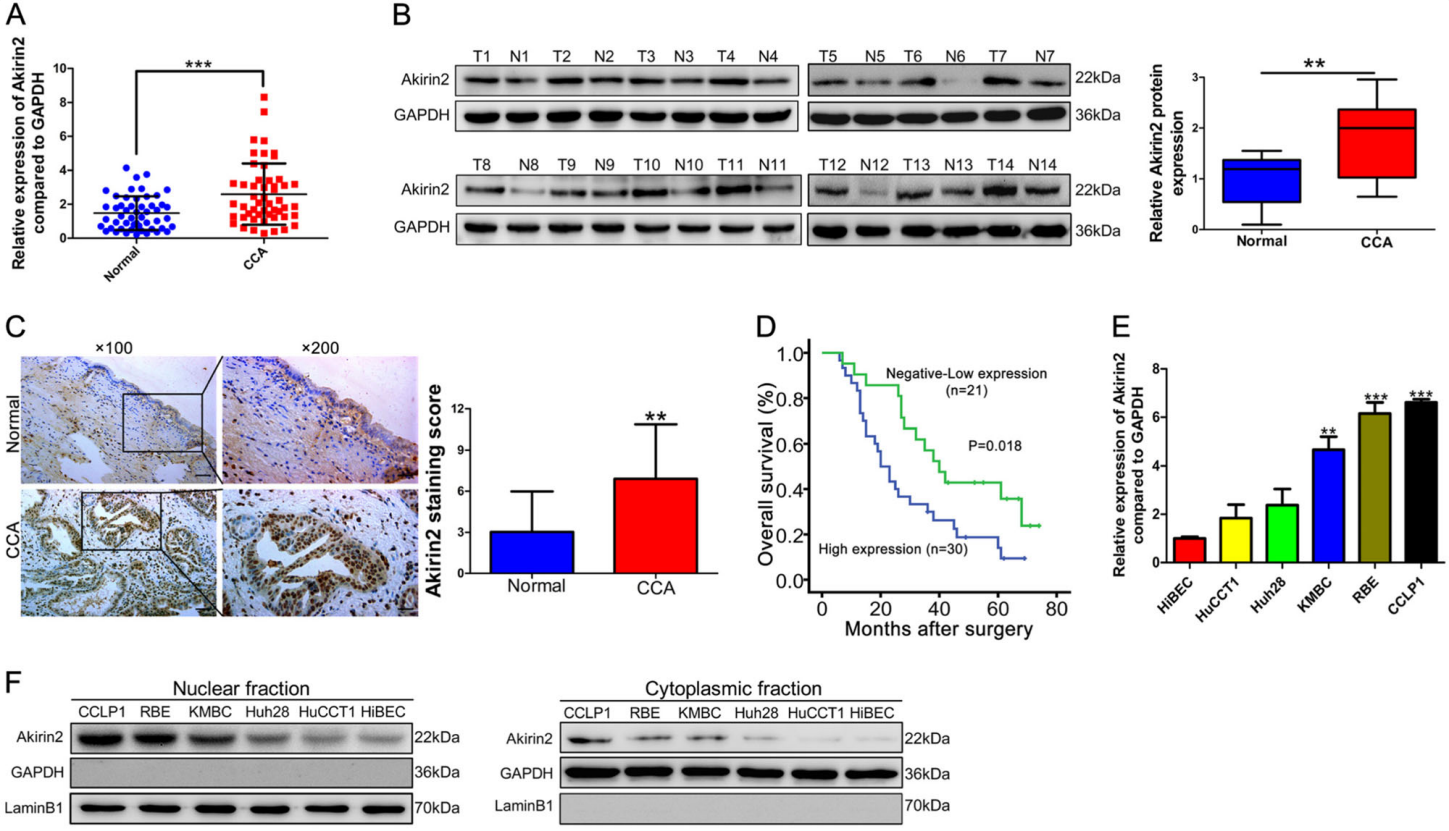
Akirin2 is upregulated in human cholangiocarcinoma (CCA) and predicts a poor prognosis. **a** Akirin2 mRNA expression was markedly elevated in CCA tissues relative to their normal counterparts determined by RT-qPCR. **b** Western blot analysis was employed to confirm the expression of Akirin2 in human CCA compared with the adjacent normal tissues. T tumors, N adjacent normal tissues. **c** Representative images of Akirin2 immunohistochemistry (IHC) staining in normal bile duct tissues and CCA tissues. **d** Kaplan–Meier analysis indicating the correlation between the prognosis of CCA patients and the high/low expression of Akirin2. **e** Relative Akirin2 mRNA levels in human intrahepatic biliary epithelial cell (HiBEC) and CCA cell lines by RT-qPCR. **f** Western blot analysis of Akirin2 in HiBEC and five CCA cell lines were performed on separated cell fractions: the nuclear fraction (left blot) and the cytoplasmic fraction (right blot). **P < 0.01; ***P < 0.001. Data are shown as mean ± SD of at least three independent experiments

We first conducted reverse transcription-quantitative polymerase chain reaction (RT-qPCR) to investigate Akirin2 transcription levels in 51 paired human CCA tissue specimens and their corresponding nontumorous tissue samples. The results showed that Akirin2 mRNA expression was markedly elevated in CCA tissues relative to their normal counterparts (Fig. 1a), which was consistent with the results from The Cancer Genome Atlas (TCGA) (Fig. S1). An upregulated protein expression level of Akirin2 was further confirmed in 14 paired specimens by immunoblotting assays (Fig. 1b). Immunohistochemistry (IHC) data illustrated that Akirin2 was mostly localized to the nucleus of CCA cells (Fig. 1c).

Based on the IHC staining results, we grouped CCA specimens according to the staining intensity of Akirin2 as low and high. The Akirin2 expression level was consistently enhanced in CCA tissues relative to the nonmalignant tissues (Fig. 1c). Next, we explored the correlations of Akirin2 expression with CCA patients’ clinicopathological features and prognosis. We found that high Akirin2 expression was closely associated with late tumor stages (*P* = 0.024) and positive lymph node invasion (*P* = 0.047) (Table S1). Further Kaplan–Meier curves indicated that patients with enhanced Akirin2 expression exhibited a worse OS than those with low expression (Fig. 1d).

To confirm the prognostic value of Akirin2, Cox regression analyses were conducted. The results documented that overexpression of Akirin2 was identified as an independent prognostic predictor of an adverse OS in CCA patients (*P* = 0.036, Table S2). Next, we performed RT-qPCR and western blot analysis to examine Akirin2 expression in a panel of human CCA cells (HuCCT1, Huh28, KMBC, RBE, and CCLP1). We found that Akirin2 was elevated at both the mRNA and protein levels in CCA cells relative to normal human intrahepatic biliary epithelial cells (HiBEC, Fig. 1e, f). In addition, Akirin2 protein was distributed in both the nucleus and cytoplasm (Fig. 1f).

### Akirin2 is required for CCA cell proliferation, migration, and invasion in vitro

**Fig. 2:**
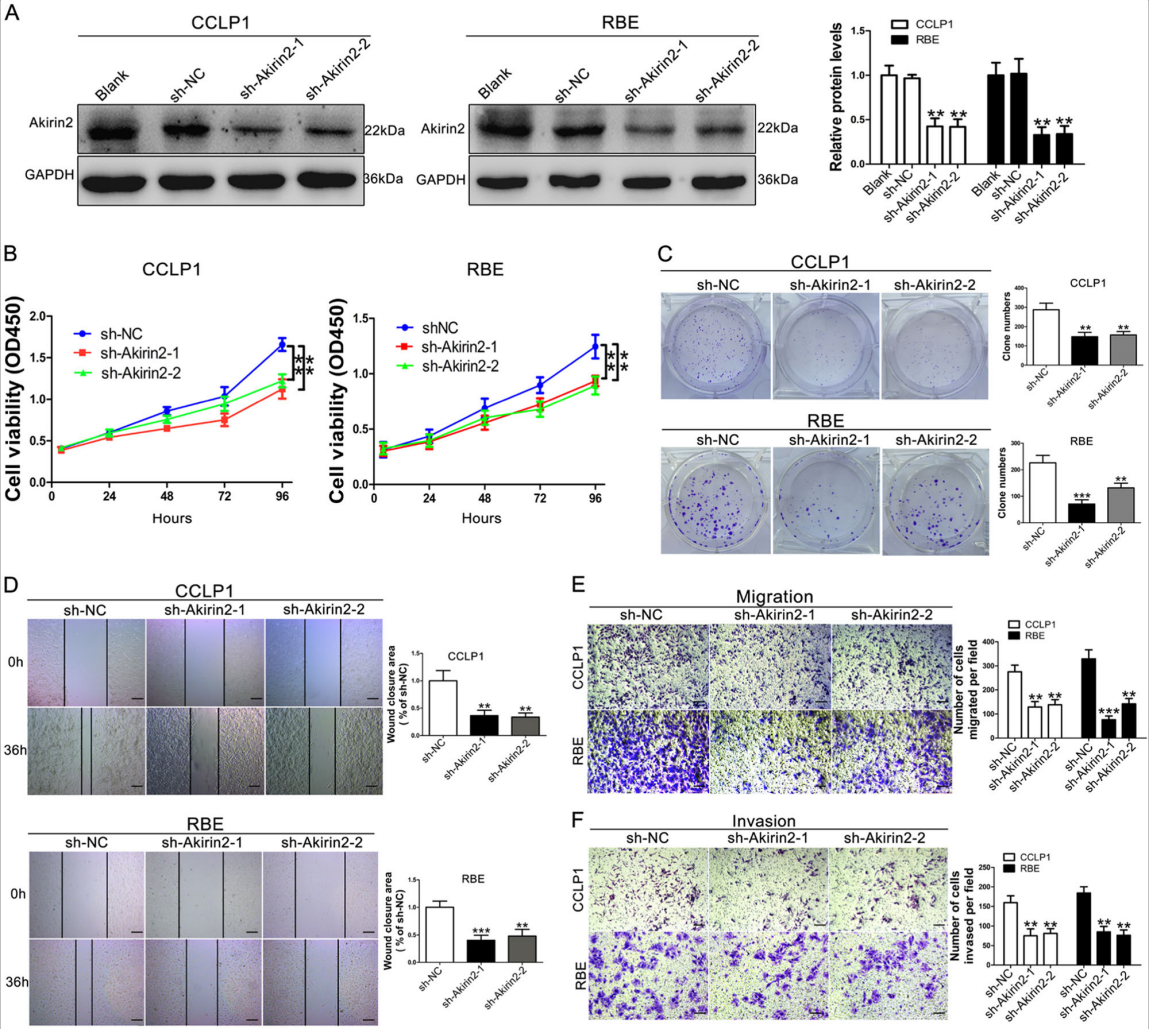
Knockdown of Akirin2 suppresses the proliferation, migration and invasion of cholangiocarcinoma (CCA) cells in vitro. **a** Western blot analysis was employed to examine the efficiency of Akirin2 knockdown. **b** Proliferation curves were determined in Akirin2 stable knockdown CCLP1 and RBE cells by cell counting kit-8 (CCK-8) assays. **c** Colony-forming abilities were measured in Akirin2 stable knockdown CCLP1 and RBE cells by clonogenic assays. **d** Wound-healing assay was performed to measure the migration ability of various cells as indicated. **e**, **f** Transwell assays were used to detect the migration and invasive capacities in Akirin2 stable knockdown CCLP1 and RBE cells. Magnification, × 40 (**d**), × 200 (**e**, **f**). Scale bar, 500 μm (**d**), 100 μm (**e**, **f**). **P < 0.001; ***P < 0.001. Data are shown as mean ± SD of at least three independent experiments

To investigate whether Akirin2 possessed a tumor-promoting function in CCA, we stably downregulated Akirin2 in high Akirin2-expressing CCLP1 and RBE cells using a lentivector carrying short hairpin RNA (shRNA). We tested the knockdown efficiencies of three different shRNAs targeting Akirin2 and selected two shRNAs (sh-Akirin2-1 and sh-Akirin2-2) that were the most effective in the knockdown study (Fig. 2a, Fig. S2A–B). Low Akirin2-expressing HuCCT1 cells were selected for upregulation of Akirin2 and the ectopically overexpressed efficiency was confirmed through comparison with the empty vector by western blotting (Fig. S3A–B).

To explore the impact of Akirin2 repression on regulating cell proliferation, we performed cell counting kit-8 (CCK-8) and clone-forming experiments. The results showed that downregulation of Akirin2 in CCLP1 and RBE cells resulted in remarkably decreased cell growth and clonogenic ability (Fig. 2b, c). Conversely, overexpression of Akirin2 in HuCCT1 significantly increased cell proliferation and colony formation ability (Fig. S3C-D). To explore whether Akirin2 was involved in cancer metastasis, we conducted wound scratch and Transwell experiments to examine the impact of Akirin2 on modifying CCA cell motility. The data showed that Akirin2 knockdown suppressed the wound closure potential in CCLP1 and RBE cells (Fig. 2d). Meanwhile, the Transwell assay showed that Akirin2 downregulated CCA cells exhibited markedly decreased capabilities of migration and invasion than the negative control cells (Fig. 2e, f). Conversely, overexpression of Akirin2 significantly enhanced the wound closure potential, migration, and invasion of HuCCT1 cells (Fig. S3E–F).

### Akirin2 modulates tumor growth and metastasis in vivo

**Fig. 3:**
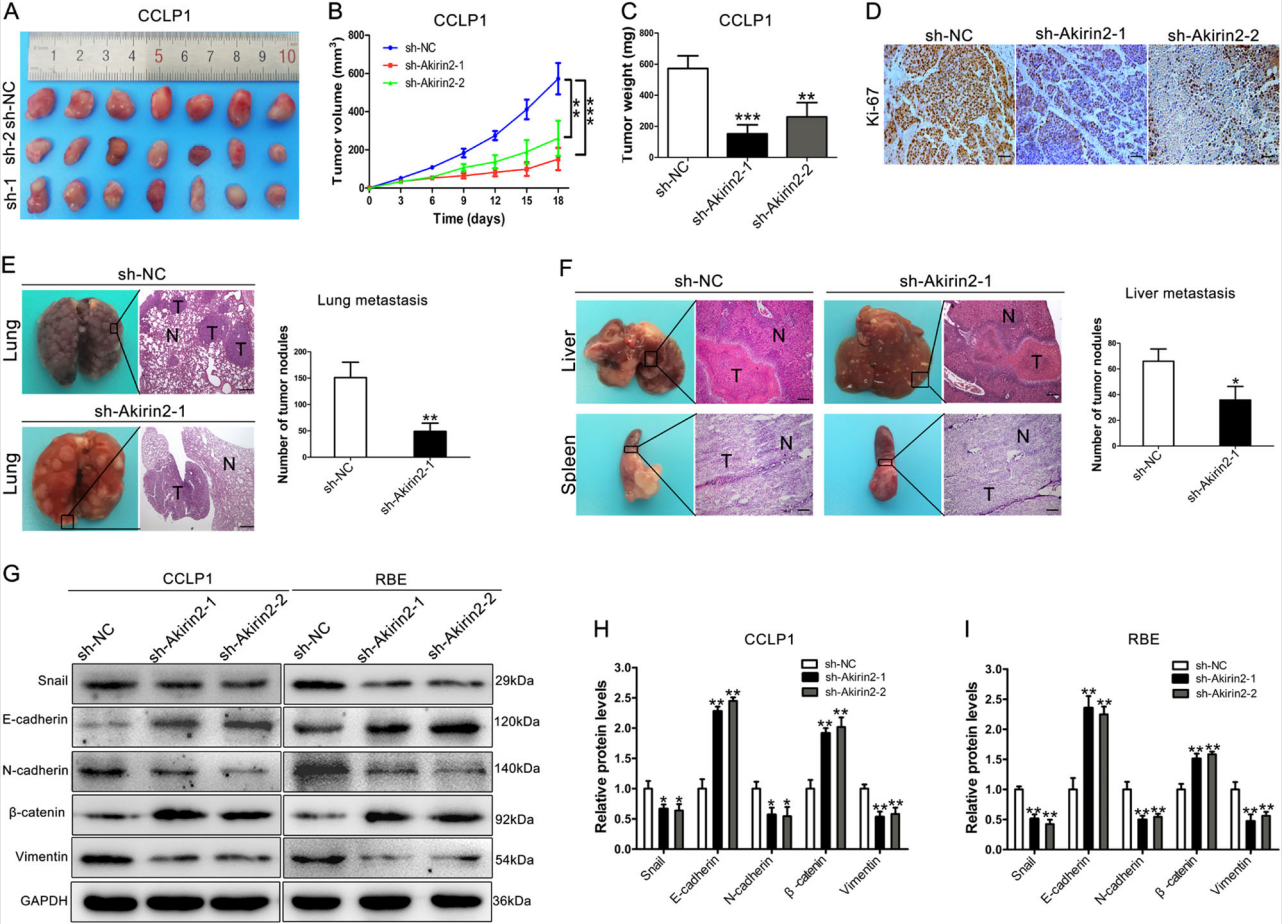
Knockdown of Akirin2 suppresses tumor growth and metastasis in vivo. **a** Xenograft tumors were generated by injecting CCLP1 cells downregulated Akirin2 or carrying a negative control vector. **b** The growth of xenograft tumors was measured by volume. **c** The tumor weight was recorded. **d** Ki67 staining of the xenograft tumors is shown. **e** Analysis of an experimental metastasis animal model was performed by injecting Akirin2 stable knockdown CCLP1 cells into the tail vein of nude mice. (Left) Representative images from each group are shown. (Right) The number of tumor nodules on lung surfaces from two groups is shown. **f** An experimental metastasis animal model was performed by injecting Akirin2 stable knockdown CCLP1 cells into the distal tip of the spleen. (Left) Representative images from each group are shown. (Right) The number of tumor nodules on liver surfaces from two groups is shown. **g**–**i** E-cadherin, β-catenin, Snail, Vimentin, and N-cadherin protein levels were detected and quantified in Akirin2 stable knockdown CCLP1 and RBE cells by western blotting. Magnification, × 40 (**d**, **e**, **f**). Scale bar, 500 μm (**d**, **e**, **f**). *P < 0.05; **P < 0.001; ***P < 0.001. Data are shown as mean ± SD of at least three independent experiments

To further validate whether Akirin2 could affect tumor growth, the aforementioned CCA cells with stable overexpression or knockdown of Akirin2 were inoculated subcutaneously into either side of nude mice. The xenograft growth was evaluated after 18 days. We found that tumors derived from Akirin2 knockdown cells grew more slowly, and the final tumor weight was markedly lower in the Akirin2 knockdown group than that in the negative control (sh-NC) group (Fig. 3a–c), which was consistent with the results in vitro. Conversely, tumor growth and tumor weight in the Akirin2-overexpressed group were significantly higher compared with the empty vector group (Fig. S4A–C). In addition, IHC results revealed that Ki67 expression was dramatically decreased in the Akirin2 knockdown group, whereas it was increased in the Akirin2-overexpressed group (Fig. 3d and Fig. S4D).

To validate whether Akirin2 downregulation also affected CCA cell metastasis in vivo, Akirin2 stable knockdown CCLP1 cells and negative control cells were inoculated into nude mice via the tail vein or distal tip of the spleen. The data showed that the numbers of metastatic nodules on the mouse lungs from the Akirin2 downregulated group were dramatically lower than in the sh-NC group (Fig. 3e). Hematoxylin and eosin (H&E) staining confirmed the differences between the two groups. In addition, as shown in Fig. 3f, Akirin2 knockdown resulted in a reduced number of metastatic nodules in the livers than in the sh-NC group. As epithelial–mesenchymal transition **(**EMT) markers are key regulators in cancer cell migration and metastasis, we asked whether Akirin2 affected the EMT process in CCA cells. Western blot analysis documented that silencing of Akirin2 increased the expression level of epithelial markers (E-cadherin and β-catenin), while decreasing the expression of mesenchymal markers (N-cadherin, Vimentin, and Snail) (Fig. 3g–i). The results were consistent with Akirin2 overexpression data (Fig. S5).

### Akirin2 regulates tumor angiogenesis in vitro and in vivo

**Fig. 4:**
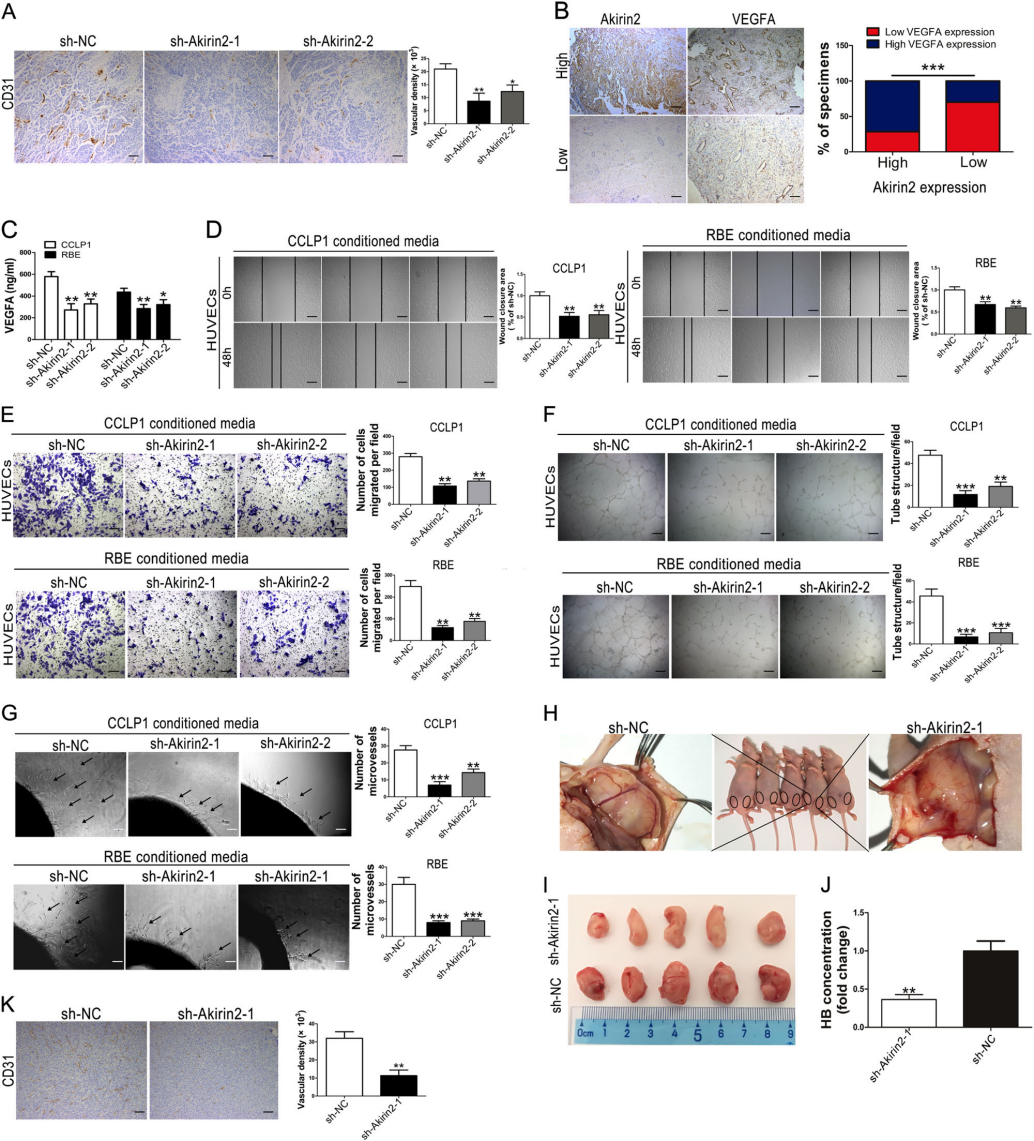
Akirin2 suppression attenuates tumor angiogenesis in vitro and in vivo. **a** CCLP1-sh-NC and CCLP1-sh-Akirin2-1 tumors were analyzed histologically using CD31-staining and CD31-staining vasculature was quantified via manually calculated average number of microvessels at × 200 magnification. **b** Expression of Akirin2 is associated with VEGFA expression levels in clinical cholangiocarcinoma (CCA) specimens. Two representative images are shown. The histogram displayed percentage of specimens showing low or high Akirin2 expression in relation to the expression levels of VEGFA. **c** The levels of VEGFA in Akirin2-sh-NC and Akirin2-sh-Akirin2 cell supernatants were detected by ELISA assay. **d**, **e** Akirin2 knockdown impaired tumor-induced HUVEC migration according to wound-healing (**d**) and Transwell migration assays (**e**). **f**, **g** Akirin2 knockdown suppressed tumor-induced HUVECs angiogenesis according to tube formation assays (**f**) and aortic ring sprouting assay (**g**). **h**–**k** Nude mice were injected on both sides of the groin subcutaneously with Matrigel mixed with the stable transfected CCLP1 cells (NC on the right side; sh-Akirin2-1 on the left side) (**h**). General morphology and color of the Matrigel plugs were recorded (**i**) and plugs were quantified for hemoglobin content (**j**). Vascular endothelial-like structures were examined by a CD31 staining (**k**). Magnification, × 40 (**a**, **b**, **d**, **f**, **k**), × 100 (**g**), × 200 (**e**). Scale bar, 500 μm (**a**, **b**, **d, f**, **k**), 200 μm (**g**), 100 μm (**e**). *P < 0.05; **P < 0.001; ***P < 0.001. Data are shown as mean ± SD of at least three independent experiments

Angiogenesis is considered to be one of the most important cancer hallmarks. Angiogenesis plays pivotal roles in tumor growth, migration, and metastasis. As Akirin2 knockdown impaired CCA cell proliferation in vitro and in vivo, we hypothesized that Akirin2 downregulation could attenuate tumor angiogenesis. Therefore, we performed CD31 IHC staining of the aforementioned subcutaneous xenograft. The IHC results indicated that the intensity of the CD31-positive microvessels was strikingly inhibited in Akirin2 downregulated group compared with the sh-NC group (Fig. 4a).

Considering VEGFA functions as a key regulator in the process of tumor angiogenesis, we then explored whether there was an association between the expression of Akirin2 and VEGFA. As shown in Fig. 4b, expression of VEGFA was strong in 21 of 30 (70%) samples with high expression of Akirin2. Conversely, high VEGFA expression was only found in 6 of 21 (28.6%) specimens expressing low levels of Akirin2. We further examined the VEGFA levels in the sh-Akirin2-1, sh-Akirin2-2 and sh-NC cell supernatants using an enzyme-linked immunosorbent assay (ELISA) assay. The data demonstrated that a lower level of VEGFA was observed in Akirin2 downregulated CCA cell supernatants than in the supernatants from sh-NC cells (Fig. 4c). Concurrently, overexpressed Akirin2 induced a significantly increased VEGFA level (Fig. S6A).

To validate that Akirin2 is a potential angiogenesis driver in CCA cells, conditioned media derived from Akirin2 overexpressing and downregulated CCA cells were used to examine their effects on the ability of human umbilical vein endothelial cells (HUVECs) to migrate and form tubes. The results showed that medium conditioned by Akirin2 downregulated cells exhibited a lower activity to promote HUVECs migratory capacity during wound-healing, Transwell assays and tube formation in Matrigel compared with the conditioned media derived from sh-NC cells (Fig. 4d–f). Conversely, migration and tube formation were enhanced by the conditioned media from Akirin2 overexpressing HuCCT1 cells (Fig. S6B–C). To further investigate the pro-angiogenic properties of Akirin2, we established ex vivo and in vivo angiogenesis models. The data indicated that conditioned media from Akirin2 downregulated CCA cells markedly inhibited microvessel sprouting from aortic rings, whereas conditioned media from Akirin2 overexpressing HuCCT1 cells enhanced microvessel sprouting from aortic rings (Fig. 4g, S6D). Next, we conducted a Matrigel plug assay to confirm the influence of Akirin2 on VEGFA-induced angiogenesis. Consistent with the previous results, the number of inguinal vessel branches in the Akirin2 knockdown group was remarkably lower than that in the sh-NC group (Fig. 4h, i). Furthermore, hemoglobin concentrations in the Matrigel plugs were tested after Matrigel dissolution. The data revealed that the Akirin2 knockdown group also exhibited decreased hemoglobin levels compared with the sh-NC group (Fig. 4j). CD31 IHC staining showed that Akirin2 knockdown decreased microvessel density in the Matrigel plug (Fig. 4k).

### Akirin2 stimulates VEGFA expression via activating the IL-6/STAT3 signaling pathway

**Fig. 5:**
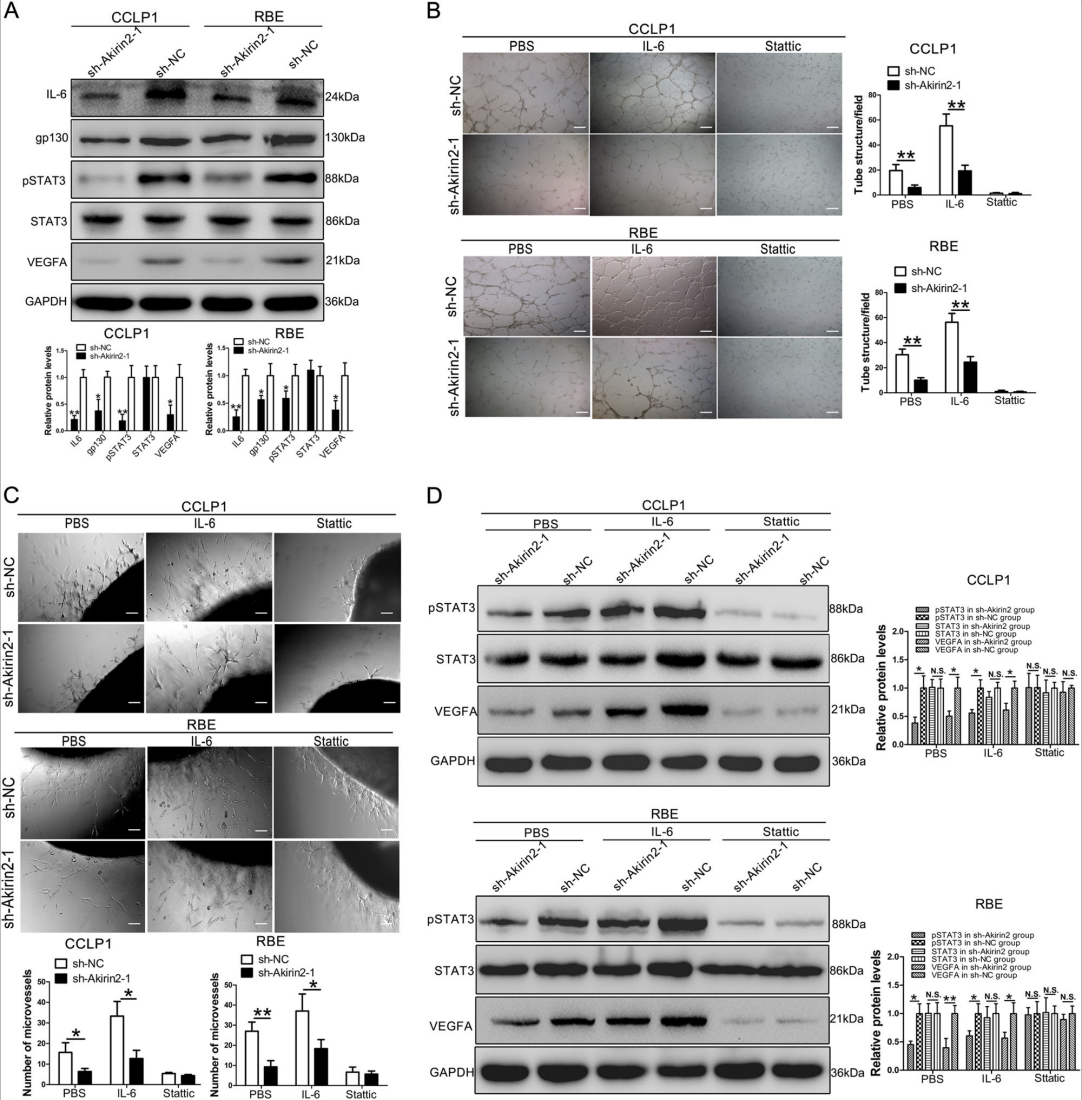
Akirin2 stimulates VEGFA expression via activating IL-6/STAT3 signaling pathway. **a** The levels of IL-6, gp130, STAT3, pSTAT3, and VEGFA in Akirin2-sh-NC and Akirin2-sh-Akirin2 cells were detected by western blot. **b**, **c**The formation of capillary-like structures was obviously promoted by Akirin2. The co-culture with IL-6 (20 ng/ml) or stattic (10 μM) was also evaluated. **d** The western blot analysis of the expression of the proteins pSTAT3, STAT3, and VEGFA post co-culture with IL-6 (25 ng/ml, 30 min) or stattic (10 μM, 2 h). Magnification, × 40 (**c**), × 100 (**d**). Scale bar, 500 μm (**c**), 200 μm (**d**). *P < 0.05; **P < 0.001. Data are shown as mean ± SD of at least three independent experiments

It has been reported that Akirin2 is involved in the NF-κB signaling pathway, and is essential for the production of IL-6 in mice^13^. We speculated that Akirin2 could also regulate IL-6 expression in CCA. Therefore, we examined IL-6 expression by western blot analysis in Akirin2 downregulated and overexpressing CCA cells. The results indicated that the expression of IL-6 in Akirin2 knockdown cells was markedly reduced (Fig. 5a), whereas it was upregulated in Akirin2 overexpressing CCA cells (Fig. S6E). It is well-known that the IL-6/STAT3/VEGFA axis plays a key role in tumor angiogenesis^22,23^. We hypothesized that Akirin2 controlled VEGFA expression via the IL-6/STAT3 axis. Western blot analysis revealed that Akirin2 knockdown dramatically decreased the expression of pSTAT3 and VEGFA (Fig. 5a). Meanwhile, the level of pSTAT3 and VEGFA in Akirin2 overexpressing cells was higher than that in the empty vector group (Fig. S6E). In addition, the expression of gp130, which was a signal-transducing subunit shared by the receptors for the IL-6 family of cytokines was significantly decreased in Akirin2 knockdown cells, whereas it was increased in Akirin2 overexpressing cells (Fig. 5a, Fig. S6E).

Next, we further performed tube formation and aortic ring sprouting assays using Akirin2 knockdown conditioned medium incubation with either phosphate buffered saline (PBS)  (control), recombinant IL-6 (20 ng/ml, 30 min) or stattic (10 μM, 2 h). Tube-forming experiment illustrated that after treatment with the recombinant IL-6, capillary-like structures were not completely restored in response to media from Akirin2 knockdown cells compared with sh-NC cells (Fig. 5b). In addition, the activity of tube formation was impaired by stattic (10 μM), and no difference was found between the Akirin2 knockdown group and the sh-NC group for both the CCLP1 and RBE cells (Fig. 5b). The results from the aortic ring sprouting assay were consistent with that from the tube formation assay (Fig. 5c). To confirm the results mentioned above, we used recombinant IL-6 and stattic to stimulate or inhibit the phosphorylation of STAT3. The data demonstrated that the expression of pSTAT3 was not completely rescued by incubation with IL-6 (Fig. 5d). pSTAT3 and VEGFA expression in the stattic treatment group were remarkably compromised in both the Akirin2 knockdown cells and sh-NC cells compared with that in the PBS treatment group, and no significant difference was found between the Akirin2 knockdown cells and the sh-NC cells (Fig. 5d).

### Akirin2 expression is negatively controlled by miR-490-3p in human CCA

**Fig. 6:**
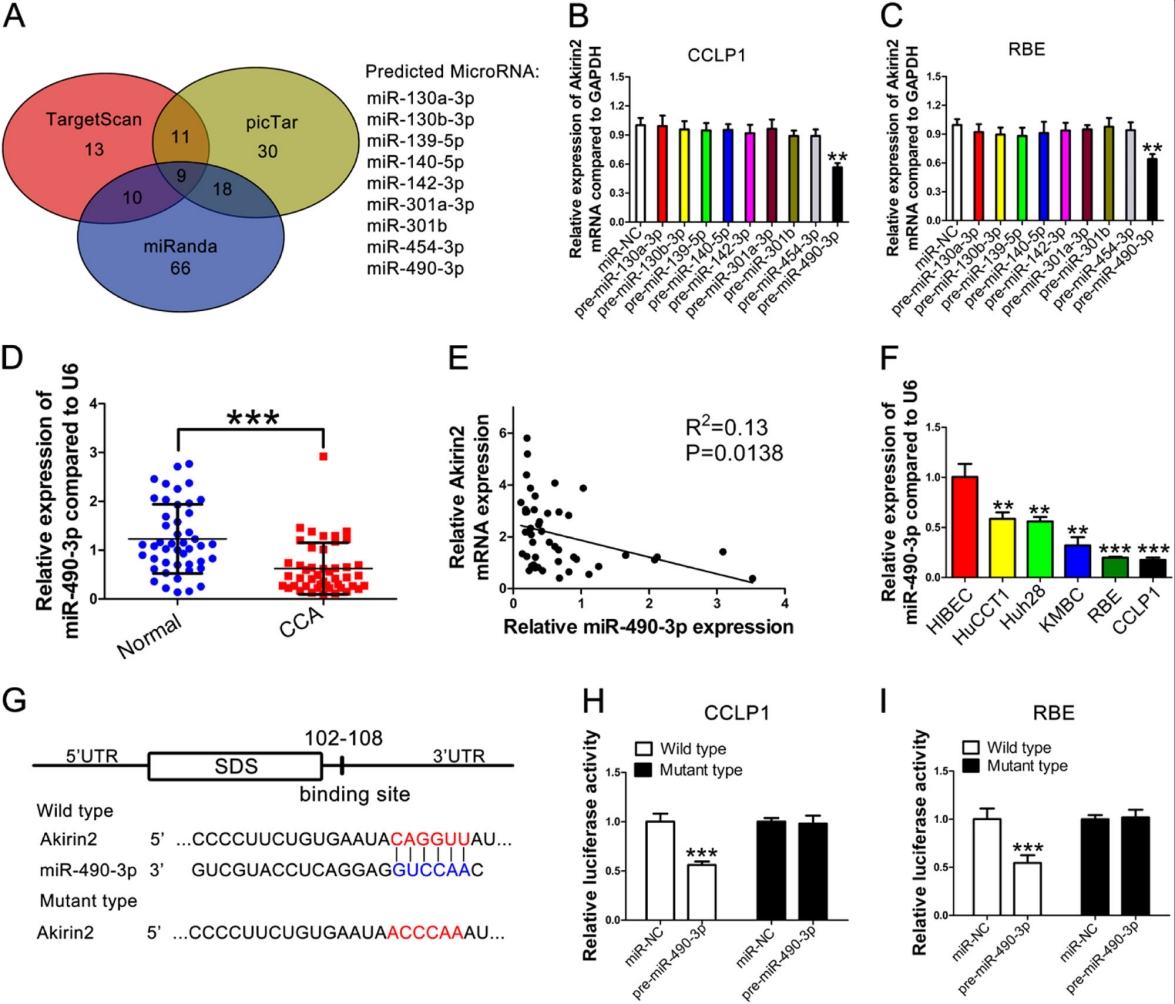
Akirin2 expression is negatively controlled by miR-490-3p in human cholangiocarcinoma (CCA). **a** Venn diagram indicates the number of predicted mirRNAs in three bioinformatic algorithms. **b, c** Akirin2 mRNA was detected by RT-qPCR after tranfecting the predicted miRNAs mimics in CCLP1 (**b**) and RBE cells (**c**). **d** Relative expression levels of miR-490-3p in adjacent normal tissues and CCA tissues. **e** Correlation between miR-490-3p expression and Akirin2 expression in clinical samples. **f** Relative miR-490-3p levels in HiBEC and CCA cell lines by RT-qPCR. **g** The putative binding site of the 3′-UTR of Akirin2 and miR-490-3p was shown. **h**, **i** Luciferase reporter assay. CCLP1 and RBE cells were co-transfected with a luciferase construct fused with the wild-type or site mutant 3′-UTR of Akirin2 and pre-miR-490-3p or miR-NC. Luciferase activity is reported relative to that of Renilla. **P < 0.001; ***P < 0.001. Data are shown as mean ± SD of at least three independent experiments

Next, we investigated the possible relationship between miRNAs deregulation and Akirin2 overexpression in CCA. Three bioinformatics databases (TargetScan, picTar, and miRanda) were used to predict the potential miRNAs interacting with the 3′-UTR of the Akirin2 mRNA. Nine miRNAs were identified in all three databases (Fig. 6a). Next, we overexpressed all nine miRNAs by transfecting the corresponding mimics to investigate their effect on Akirin2 mRNA expression in CCLP1 and RBE cells. The results indicated that only miR-490-3p significantly compromised the levels of Akirin2 mRNA, suggesting that overexpression of Akirin2 might be attributable to the low expression of miR-490-3p (Fig. 6b, c). After that step, we explored the level of miR-490-3p in 48 paired human CCA tissue samples and their matched normal tissue specimens. As expected, the level of miR-490-3p was dramatically downregulated in CCA tissues relative to the nontumorous tissue samples (Fig. 6d), and the Akirin2 mRNA was inversely correlated with miR-490-3p expression in the CCA tissues (Fig. 6e). Furthermore, a lower miR-490-3p level was observed in CCA cell lines relative to HiBEC (Fig. 6f).

To investigate the binding ability between miR-490-3p and the 3′-UTR of Akirin2, a luciferase reporter assay was conducted using wild-type and mutant versions of the Akirin2 3′-UTR (Fig. 6g). Figures 6h, i show the results that ectopic miR-490-3p expression evidently repressed the activity of luciferase in CCLP1 (Fig. 6h), RBE (Fig. 6i) and HuCCT1 (Fig. S7) cells. Moreover, a mutant reporter of the Akirin2 3′-UTR disrupted the suppressive effect of miR-490-3p. Taken together, the above findings documented that the upregulation of Akirin2 in CCA was partially attributed to miR-490-3p downregulation.

### Akirin2 is involved in the miR-490-3p-mediated regulation of CCA migration and angiogenesis

**Fig. 7:**
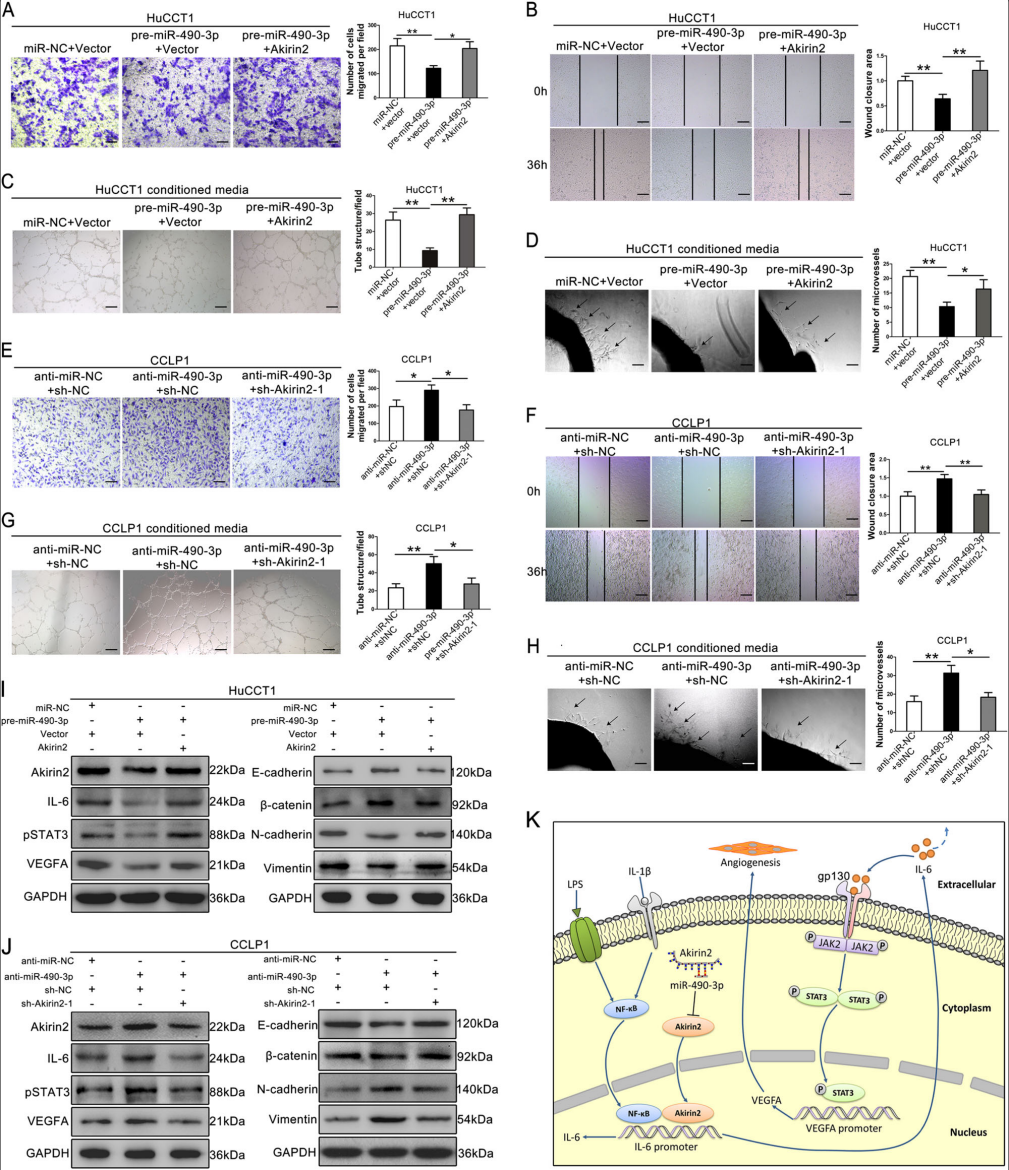
Akirin2 is involved in the miR-490-3p-mediated regulation of cholangiocarcinoma (CCA) migration and angiogenesis. **a**, **b** Migration ability was partly restored in pre-miR-490-3p treated HuCCT1 cells after re-expression of Akirin2. **c**, **d** Enforced expression of Akirin2 partially reversed the angiogenic activity of HuCCT1 cells with miR-490-3p overexpression. **e**, **f** Migration ability was partly compromised in anti-miR-490-3p treated CCLP1 cells after knockdown of Akirin2. **g**, **h** Akirin2 knockdown impaired the ability of tube formation and aortic ring sprouting of CCLP1 cells with miR-490-3p inhibition. **i** Western blot analysis of the levels of pSTAT3, STAT3, VEGFA, and EMT marker in HuCCT1 cells transfected with equal doses of miR-NC plus vector, pre-miR-490-3p plus vector, pre-miR-490-3p plus Akirin2 overexpression plasmid. **j** Western blot analysis of the levels of pSTAT3, STAT3, VEGFA, and EMT marker in CCLP1 cells transfected with equal doses of anti-miR-NC plus sh-NC plasmid, anti-miR-490-3p plus sh-NC plasmid, anti-miR-490-3p plus Akirin2 downregulation plasmid. **k** Schematic model of Akirin2-induced tumor angiogenesis in CCA cells. Akirin2 induced IL-6 expression by activating NF-κB signaling pathway. Akirin2-induced IL-6 triggered the activation of IL-6/STAT3/VEGFA signaling pathway, resulting in enhanced tumor angiogenesis. Magnification, × 40 (**b**, **c**, **f**, **g**), × 100 (**d**, **h**), × 200 (**a**, **e**). Scale bar, 500 μm (**b**, **c**, **f**, **g**), 200 μm (**d**, **h**), 100 μm (**a**, **e**). *P < 0.05; **P < 0.001. Data are shown as mean ± SD of at least three independent experiments

To further determine whether miR-490-3p influenced CCA cells by directly targeting Akirin2, rescue experiments were conducted in high Akirin2-expressing CCLP1 cells and low Akirin2-expressing HuCCT1 cells. We co-transfected HuCCT1 cells with the following synthetic RNAs/plasmids: (1) miR-NC and vector, (2) pre-miR-490-3p and vector, (3) pre-miR-490-3p and an Akirin2 overexpression plasmid. As expected, HuCCT1 cells co-transfected with pre-miR-490-3p and Akirin2 exhibited remarkably improved capabilities of migration (Fig. 7a, b) and angiogenesis (Fig. 7c, d) compared with the cells transfected with pre-miR-490-3p alone. The results showed that overexpression of Akirin2 efficiently restored the pre-miR-490-3p-medieted tumor-suppressive effects, as represented by enhanced cell migration and angiogenesis of CCA cells. We subsequently co-transfected CCLP1 cells with the following synthetic RNAs/plasmids: (1) anti-miR-NC and sh-NC plasmid, (2) anti-miR-490-3p and sh-NC plasmid, (3) anti-miR-490-3p and Akirin2 downregulation plasmid. The data demonstrated that CCLP1 cells co-transfected with anti-miR-490-3p and sh-Akirin2-1 showed remarkably decreased capabilities of migration (Fig. 7e, f) and angiogenesis (Fig. 7g, h) compared with the cells transfected with anti-miR-490-3p alone. These findings indicated that the effect of anti-miR-490-3p in promoting cellular migration and angiogenesis was significantly reversed by cotransfection of the Akirin2 knockdown plasmid. Consistent with biological phenotypes, re-expression of Akirin2 in HuCCT1 cells rescued the pre-miR-490-3p-mediated alteration in the IL-6/STAT3 pathway and EMT markers (Fig. 7i). Conversely, knockdown of Akirin2 in CCLP1 cells also reversed the anti-miR-490-3p-mediated alteration in the IL-6/STAT3 pathway and EMT signature (Fig. 7j). Collectively, the above data indicated that miR-490-3p could suppress migration and angiogenesis in human CCA cells by inhibiting Akirin2.

## Discussion

In the current study, we demonstrated that Akirin2 expression was markedly elevated in CCA tissues and cell lines relative to the normal tissues and cell lines. This progressively upregulated expression was positively associated with poor outcomes in CCA patients. Moreover, Akirin2 levels were positively correlated with VEGFA expression in CCA tissues, indicating that Akirin2 might promote tumor angiogenesis in human CCA. Consistently, we found that Akirin2 overexpression upregulated, whereas endogenous Akirin2 knockdown eliminated VEGFA expression and angiogenesis through the IL-6/STAT3 signaling pathway, and thus facilitated tumor growth, invasion, and metastasis. In addition, miR-490-3p was identified as a negative regulator of Akirin2 expression in human CCA tissue samples and cells, and regulated cell migration and angiogenesis by silencing Akirin2.

In recent years, the pivotal roles of EMT in CCA invasion and metastasis have been well documented^24–26^. Recently, an interesting experiment demonstrated that mutations in E-cadherin, Kras, and TGFβR2 cooperatively resulted in CCA tumorigenesis^27^. Our study further showed that knockdown of Akirin2 resulted in an elevated E-cadherin expression and decreased expression of vimentin and N-cadherin. Accumulating evidence has shown that Snail is a pivotal transcriptional element of EMT, which promotes vimentin expression and decreases the expression of E-cadherin (in)directly^28^. Our previous study demonstrated that the SPRY4-IT1/Snail/E-cadherin pathway played an important function in promoting CCA migration and invasion^29^. In the current study, we found that Snail expression was correlated with Akirin2 expression alteration in CCA cells. Therefore, it is possible that Akirin2 induced EMT by regulating Snail. Further study is needed to confirm this hypothesis.

Sustained angiogenesis is an essential component of tumor unrestrained growth and metastasis. Therefore, identifying a promising anti-angiogenic therapy target is thought to be an effective treatment for cancers. Currently, accumulated studies have reported several angiogenesis-related regulators in CCA^30–32^. In this study, we reported for the first time that Akirin2 is a novel angiogenesis driver that indirectly regulated VEGFA expression in human CCA. It has previously been reported that Akirin2 is a downstream effector of the NF-κB signaling pathway and this pathway leads to the production of IL-6 in mice^13^. Our finding revealed that IL-6 expression is altered significantly in response to Akirin2 expression changes, indicating that Akirin2 might also regulate IL-6 expression through the NF-κB pathway in the context of CCA. However, this hypothesis needs further experiments to validate it. STAT3 hyperactivation is an important hallmark in cancer initiation and progression, which can transcriptionally regulate the expression of a number of genes to enhance cell growth, migration, invasion, and angiogenesis. In addition, IL-6 is known as the most well-established upstream activator of STAT3^33–35^. We speculated that Akirin2 might enhance CCA angiogenesis via activating the IL-6/STAT3/VEGFA signaling pathway (Fig. 7k). Consistent with our prediction, the in vitro experiments indicated that Akirin2 markedly increased the phosphorylation of STAT3, resulting in upregulation of VEGFA. However, the rescue experiments showed that the usage of recombinant IL-6 did not completely rescue the Akirin2 knockdown phenotype. We speculated that Akirin2 acted as a transcriptional cofactor that might also (in)directly regulate a key element of the IL-6/STAT3/VEGFA signaling pathway. As expected, we found that the expression of gp130 was significantly decreased after Akirin2 knockdown, indicating that Akirin2 enhances angiogenesis in CCA through activating the IL-6/gp130/STAT3/VEGFA axis. The underlying molecular mechanisms of Akirin2 in regulating gp130 expression need further experiments to verify.

With regard to the molecular mechanism of upregulation of Akirin2, we focused on miRNAs deregulation that might negatively regulate Akirin2 expression. Previous studies demonstrated that miRNAs plays important roles in promoting or suppressing cancer development. Here, we reported that miR-490-3p could function as a tumor suppressor by targeting the 3′-UTR of Akirin2 mRNA to inhibit CCA cell migration and tube formation. In line with our findings, miR-490-3p was found to inhibit vasculogenic mimicry and invasion by targeting the 3′-UTR of vimentin in clear cell renal cell carcinoma cells^36^. However, it is uncertain whether miR-490-3p also directly targets the 3′-UTR of vimentin and thus suppresses migration and tube formation in CCA cells. Further studies are required to investigate these details. The deregulation of miRNAs plays a vital role in the EMT process during tumor invasion and metastasis^37^. For example, miR-490-3p reverses EMT by repressing the ERGIC3 in hepatocellular carcinoma^38^. Another study showed that miR-490-3p inhibited EMT by regulating FRAT1 in colorectal cancer^39^. Accordingly, our study further indicated that miR-490-3p could inhibit EMT by negatively regulating Akirin2 in CCA. Considering that a number of miRNAs have been used as treatment targets and evaluated in clinical trials^40^, miR-490-3p overexpression sheds new light on treatments for CCA patients. However, these therapeutic strategies require further study using patient-derived tumor xenograft mouse models.

## Conclusions

Our results showed that Akirin2 was overexpressed in human CCA cell lines and tumor tissues and that Akirin2 overexpression could promote CCA cell proliferation, migration, invasion, and angiogenesis both in vivo and in vitro. We further elucidated that Akirin2 could promote angiogenesis via the IL-6/STAT3/VEGFA signaling pathway. We also identified that miR-490-3p promotes CCA cell migration and angiogenesis by directly targeting Akirin2. These results study suggest that downregulation of Akirin2 and/or overexpression of miR-490-3p may represent promising therapeutic strategies for CCA patients.

## Methods

### Patient selection and tissue specimens

Between January 2012 and January 2015, 51 paired CCA tissue samples and corresponding noncancerous tissues were acquired from the patients who underwent radical surgery at the Second Affiliated Hospital of Harbin Medical University. All of the patients signed the informed consent before the study. Fresh specimens were snap-frozen and preserved in liquid nitrogen. The criteria of the included specimens were consistent with our previous study^29^. No patient received chemotherapy, radiotherapy, or immunotherapy before the surgical procedure. The project was authorized by the Ethics Review Committees of the Second Affiliated Hospital of Harbin Medical University.

### RT-qPCR

Total RNA from CCA tissue specimens and cultured cells was isolated by TRIzol (Sigma, MO, USA) and then 1 μg of RNA was applied to synthesize the complementary DNA (cDNA) with a Transcriptor First Strand cDNA Synthesis Kit (Roche, Germany). In addition, a miRcute Plus miRNA First-Strand cDNA Synthesis Kit (TIANGEN, Beijing) was used for miRNAs cDNA synthesis. Specific gene expression was detected by using the FastStart Universal SYBR Green Master Kit (Roche, Germany) and miRNAs expression was examined by using the miRcute Plus miRNA qPCR Detection Kit (TIANGEN, Beijing). Sequences of all of the genes and miRNAs special primers are listed in Table S1. GAPDH and U6 were used for internal control of the expression of mRNA and miRNAs, respectively. The relative expression data were normalized and calculated by using the equation 2 ^-△△CT^.

### IHC assay and quantification of microvessel density

Human CCA tissues and mouse tumor tissues were fixed in paraformaldehyde immediately after resection and stained as our previous study described^41^. The slides were incubated with the primary antibodies against Akirin2, (1:150, SAB, Jiangsu, China), VEGFA, (1:100, Abcam, Cambridge, MA, USA), CD31, (1:100, Cell Signaling Technology, Danvers, MA, USA), and Ki67 (1:500, Abcam, Cambridge, MA, USA). The IHC score was calculated by multiplying the stain intensity (0 = no staining, 1 = weak staining, 2 = moderate staining, 3 = strong staining) and the scores of the number of positive tumor cells (0 = <5%, 1 = from 6 to 25%, 2 = from 26 to 50%; 3 = from 51 to 75%; and 4 = higher than 75%). The kappa statistic was calculated between the two pathologists in this study, and the kappa value was 0.80. High expression of Akirin2 was defined as an IHC score higher than 6.

Microvessels in tumor sections were examined with CD31 staining. Three areas of highest vascular density in each slide was identified at ×25 magnification, and then manually calculated average number of microvessels at ×200 magnification.

### Immunoblotting analysis

Immunoblotting assays were conducted as previously described in our earlier study^42^. The following primary antibodies were applied for western blots in this study: Akirin2 (1:500), GAPDH (1:10000), LaminB1(1:5000), VEGFA (1:500), E-cadherin (1:10000), N-cadherin (1:5000), and Vimentin (1:2000) (Abcam, Cambridge, MA, USA); Snail (1:1000), β-catenin (1:1000), gp130 (1:1000), IL-6 (1:1000), STAT3 (1:1000), and pSTAT3 (1:1000) (CST, Danvers, MA, USA).

### Cell culture

RBE and HUVECs were commercially obtained from the Cell Bank of the Chinese Academy of Sciences (Shanghai, China). CCLP1, KMBC, HiBEC, HuCCT1, and HuH28 were kindly provided by Prof. LX Liu, from the First Affiliated Hospital of Harbin Medical University. RBE, CCLP1, HiBEC, HuCCT1, and HuH28 were maintained in RPMI-1640 (Gibco, Grand Island, NY, USA) containing 10% fetal bovine serum (FBS, HyClone, Logan, UT, USA) at 37 °C in a 5% CO_2_ atmosphere. HUVECs were maintained in endothelial cell medium (ScienCell Research Laboratories) containing 10% FBS.

### Construction of stable CCA cell lines

Based on the GenBank information of Akirin2 (NM_018064), the sequences of the three shRNAs targeting Akirin2 (sh-Akirin2-1, sh-Akirin2-2, and sh-Akirin2-3) and negative control-shRNA (sh-NC) sequences are shown in Table S1. Lentiviral vectors encoding sh-Akirin2 were constructed using the GV493 vector (Genechem lnc., Shanghai, China). Sh-NC was used to monitor nonspecific responses caused by heterologous shRNA. Lentiviral vectors encoding Akirin2 were generated using the GV492 vector (Genechem lnc., Shanghai, China) and designated as LV-Akirin2. The empty vector (LV-vector) was used as a negative control. Lentiviral infection was conducted following the instructions of the manufacturer. Then, the selection of single-cell clonal isolates was performed by using puromycin for 2–4 weeks.

### Transient transfection

Overexpression of miRNAs in this study was achieved via transfecting CCA cells with miRNAs mimics. miR-490-3p downregulation was achieved by transfecting CCA cells with an miR-490-3p inhibitor. The mimics, inhibitor, and their corresponding control oligonucleotides were acquired from GenePharma (Shanghai, China). Lipofectamine 3000 reagent (Thermo Fisher Scientific, USA) was used for transient transfection following the protocol of the manufacturer. The treated cells were collected at 48 or 72 h after transfection for subsequent RT-qPCR and western blot analysis.

### CCK-8 and colony-forming experiments

CCK-8 (Dojindo, Japan) was conducted to determine the ability of CCA cell proliferation. A density of 4 × 10^3^ cells per well were seeded in 96-well plates, then 10 μl of CCK-8 reagent was added and the 96-well plates were maintained for 2 h at 37 °C. At 6, 24, 48, 72, and 96 h, the cells were measured at a wavelength of 450 nm using an ELISA reader (Tecan, Switzerland). For colony formation assays, treated cells were plated in six-well plates at a concentration of 1000 cells per well. At 10 days after plating, the cells were fixed and stained. The visible colonies were counted using a microscope.

### Conditioned media and ELISA

After CCLP1 and RBE cells (both negative control plasmid cells and Akirin2 shRNA cells) or the HuCCT1 cells (both empty vector cells and Akirin2-overexpressed cells) grew to approximately 80% confluence, the cells were cultured in RPMI-1640 for another 24 h. The conditioned media were collected and centrifuged at 2000 rpm for 10 min, and then filtered via a 0.22 µm filter. Tumor-derived VEGFA concentration in the conditioned media was examined by ELISA using an ELISA Kit (Abcam, USA) following the directions of the manufacturer.

### Wound scratch assay

Wound scratch experiments were used to investigate the migration capacity of CCA cells and HUVECs. Akirin2 downregulated or overexpressing CCA cells (including negative control cells) were planted in a 3.5 cm dish and formed a monolayer at approximately 90% confluence. Then, wounds were created by making a scratch using a pipette tip and the cells were maintained in RPMI-1640 medium. The acellular area for the CCLP1 and RBE cells were measured at 0 and 36 h. The data for the HuCCT1 cells were measured at 0 and 24 h. In another set of wound-healing experiments, HUVECs were cultured in conditioned medium from Akirin2 downregulated or overexpressing CCA cells (including negative control cells).

### Migration and invasion assays

Transwell chambers (Costar, Washington, DC, USA) coated with Matrigel (for the invasion assay) or without Matrigel (for the migration assay) were applied to further access the ability of cell invasion and migration. First, 1 × 10^5^ cells were seeded in the top chambers and incubated with RPMI-1640. Cultured medium with 10% FBS was placed in the lower champers. For HUVECs cells, conditioned medium was added in the lower chambers. After incubating for 48h at 37 °C, cells on the upper surface of the filter were eliminated. Cells on the lower surface of the filter were fixed with 4% paraformaldehyde and then stained by crystal violet. The invasive or migrated cells were counted under an inverted microscope.

### In vivo growth and metastasis study

BABL/c nude mice (6-week of age, Vital River, Beijing, China) were used for the xenograft study. The experiments were performed in accordance with the guidelines of the Animal Care and Use Committee of Harbin Medical University. To explore the tumor proliferation promoting role of Akirin2 in vivo, Akirin2 downregulated CCLP1 cells and sh-NC cells were used. The capacity for tumor growth was determined by the previously described method^29^. For the tumor metastasis assay, we established lung metastatic CCA models with 1 × 10^7^ cells injected into nude mice through the tail vein. Seven weeks after inoculation, the mice were killed, and all of the lungs were collected and stained by H&E staining. We then established liver metastatic CCA models to further evaluate the metastatic capacity of the treated CCLP1 cells. After making a 1-cm incision in the upper left lateral abdomen, the spleen was found and exposed completely. Then, 1 × 10^6^ cells were inoculated in the distal tip of the spleen. Eight weeks after injection, the liver and spleen were dissected and embedded in paraffin for H&E staining.

### Tube-forming experiment

We used 50% Matrigel (50 μl/well, BD Biosciences, CA, USA) to coat a precooled 96-well plate and it was left to polymerize at 37 °C for 30 min. Next, 3 × 10^4^ HUVECs cells were suspended in a mixture of conditioned medium (50 μl) and endothelial cell medium (50 μl) containing 10% FBS. Tube structures were photographed after incubation for 6 h at 37 °C, and analyzed with Image J (Media Cybernetics, MD, USA) as described previously^43^.

### Aortic ring sprouting assay

Thoracic aortas were obtained from 6-week old Sprague–Dawley rats. The fibroadipose tissue arround the aortas was resected and the thoracic aortas were dissected into rings of 1–2 mm. Precooled 48-well plates were coated with Matrigel. The aortic rings were embedded in the 48-well plate, and then conditioned medium was placed into the wells. At 7 days of incubation at 37 °C, the microvessels were photographed and counted according to the criteria described previously^44^.

### Matrigel plug assay

Five female nude mice were used for Matrigel plug angiogenesis assay. A total of 1 × 10^7^ cells mixed with Matrigel were injected into the bilateral ventral region. The sh-NC group cells were injected into the right groin area, whereas the sh-Akirin2-1 group cells were injected into the left groin area. Then, 10 days after injection, the mice were killed and the Matrigel plugs were dissected. The resected Matrigel plugs were then used for CD31 IHC assay and the measurement of hemoglobin concentration using Drabkin’s reagent (Sigma, USA) following the directions of the manufacturer.

### Luciferase reporter assays

The luciferase reporter plasmids containing wild-type or mutant Akirin2 3′-UTR were purchased from Genechem (Shanghai, China). For the luciferase reporter assay, CCLP1 and RBE cells were seeded in 24-well plates. Each well was transfected with 0.3 μg of firefly luciferase reporter plasmid, 0.15 μg of a Renilla expression vector (Ambion, USA), and 100 pmol of miR-490-3p mimics or the control miRNAs by using Lipofectamine 3000 transfection reagent (Invitrogen, Carlsbad, USA). After 36 h, luciferase intensity was detected by a luciferase assay kit (Promega, Madison, WI, USA).

### Data analysis

All statistical analyses were performed by GraphPad Prism 5.01 software (San Diego, CA) and SPSS 22.0 software (Chicago, IL, USA). Quantitative data were expressed as mean ± S.D. Significant differences for quantitative data were compared by two-tailed Student’s *t*-test. The *χ*^2^ test was performed to analyze the association between Akirin2 levels and clinicopathological parameters. The survival curve was drawn using Kaplan–Meier analysis with the log-rank test. A Cox proportional hazards model was used for survival analyses. *P* < 0.05 was considered to be significant.

## Supplementary information

Figure S1

Figure S2

Figure S3

Figure S4

Figure S5

Figure S6

Figure S7

Table S1

Table S2

Table S3

Supplementary figure legends
