# Supplementary figures and images for: Akirin2 is modulated by miR-490-3p and facilitates angiogenesis in cholangiocarcinoma through the IL-6/STAT3/VEGFA signaling pathway
Source: Cell Death Dis. 2019 Mar 18;10(4):262. doi: 10.1038/s41419-019-1506-4 (PMC6423123; doi:10.1038/s41419-019-1506-4)

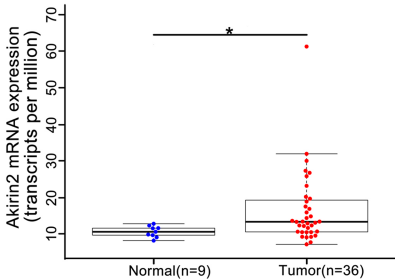

Supplement: Supplementary file 1 — Figure S1 [file 41419_2019_1506_MOESM1_ESM.pdf]

**A**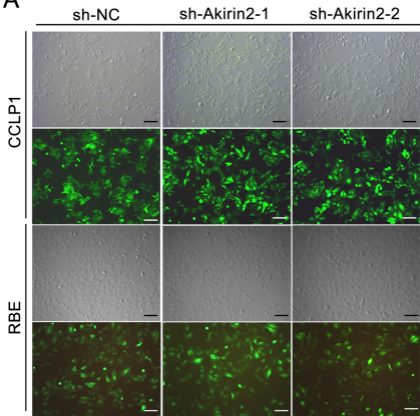**B**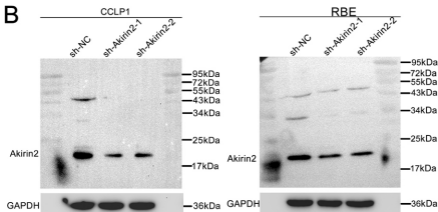

Supplement: Supplementary file 2 — Figure S2 [file 41419_2019_1506_MOESM2_ESM.pdf]

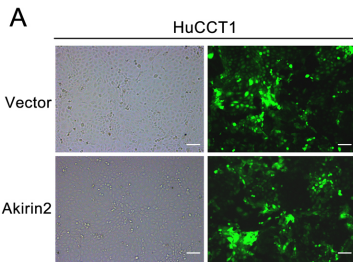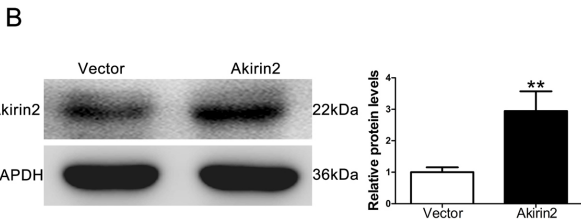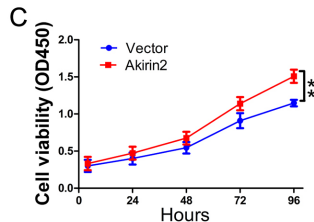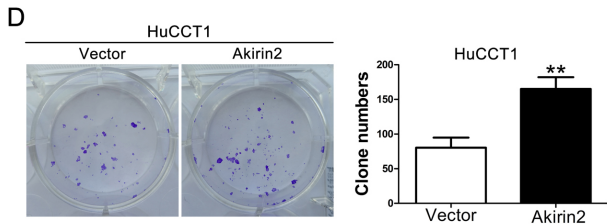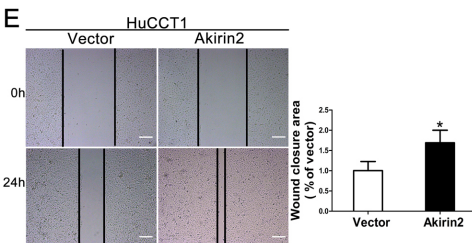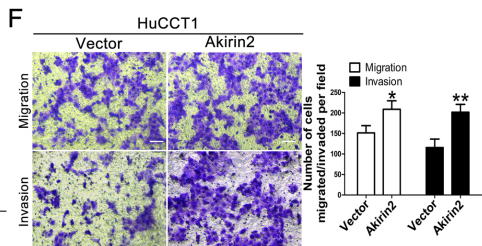

Supplement: Supplementary file 3 — Figure S3 [file 41419_2019_1506_MOESM3_ESM.pdf]

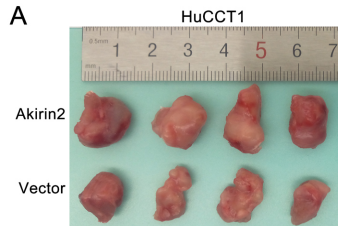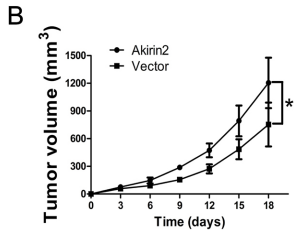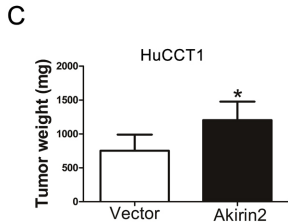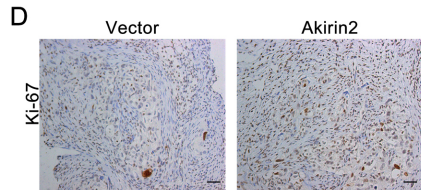

Supplement: Supplementary file 4 — Figure S4 [file 41419_2019_1506_MOESM4_ESM.pdf]

Vector

Akirin2

E-cadherin

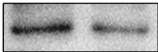

120kDa

$\beta$ -catenin

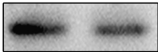

92kDa

N-cadherin

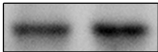

140kDa

Vimentin

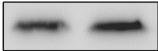

54kDa

GAPDH

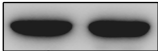

36kDa

Supplement: Supplementary file 5 — Figure S5 [file 41419_2019_1506_MOESM5_ESM.pdf]

**A**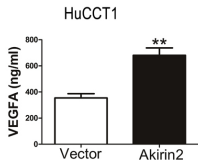**B**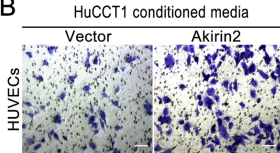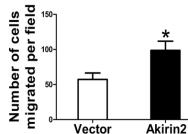**C**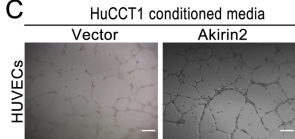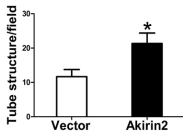**D**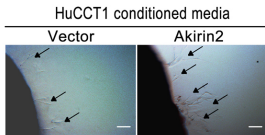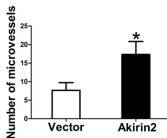**E**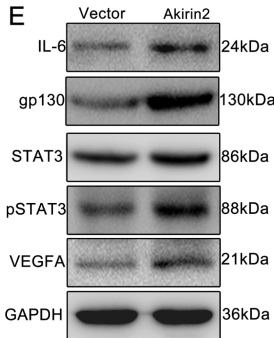

Supplement: Supplementary file 6 — Figure S6 [file 41419_2019_1506_MOESM6_ESM.pdf]

# HuCCT1

Relative luciferase activity

Wild type  
Mutant type

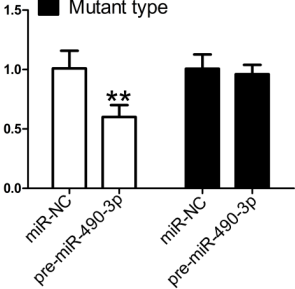

Supplement: Supplementary file 7 — Figure S7 [file 41419_2019_1506_MOESM7_ESM.pdf]
