# Supplementary material for: Akirin2 is modulated by miR-490-3p and facilitates angiogenesis in cholangiocarcinoma through the IL-6/STAT3/VEGFA signaling pathway
Source: Cell Death Dis. 2019 Mar 18;10(4):262. doi: 10.1038/s41419-019-1506-4 (PMC6423123; doi:10.1038/s41419-019-1506-4)
Supplement: Supplementary file 8 — Table S1 [file 41419_2019_1506_MOESM8_ESM.doc]

Table S1. Relationship between Akirin2 expression and clinicopathologic characteristics of CCA patients.

| Clinical characteristics | Total | Akirin2 expression | | P-value |
| --- | --- | --- | --- | --- |
|  |  | Low | High |  |
| Age |  |  |  | 0.227 |
| ≤ 60 years | 27 | 9 | 18 |  |
| ＞ 60 years | 24 | 12 | 12 |  |
| Gender |  |  |  | 0.615 |
| Male | 27 | 12 | 15 |  |
| Female | 24 | 9 | 15 |  |
| Serum CA19-9 level |  |  |  | 0.123 |
| >37 U/ml | 33 | 11 | 22 |  |
| ≤37 U/ml | 18 | 10 | 8 |  |
| Histologic differentiation |  |  |  | 0.342 |
| Well | 13 | 6 | 7 |  |
| Moderate | 23 | 7 | 16 |  |
| Poor | 15 | 8 | 7 |  |
| TNM stage |  |  |  | 0.024 |
| I-II | 22 | 13 | 9 |  |
| III-IV | 29 | 8 | 21 |  |
| Lymph node invasion |  |  |  | 0.047 |
| Present | 23 | 6 | 17 |  |
| Absent | 28 | 15 | 13 |  |
