# Supplementary material for: Akirin2 is modulated by miR-490-3p and facilitates angiogenesis in cholangiocarcinoma through the IL-6/STAT3/VEGFA signaling pathway
Source: Cell Death Dis. 2019 Mar 18;10(4):262. doi: 10.1038/s41419-019-1506-4 (PMC6423123; doi:10.1038/s41419-019-1506-4)
Supplement: Supplementary file 9 — Table S2 [file 41419_2019_1506_MOESM9_ESM.doc]

Table S2. Univariate and multivariate Cox regression analysis of Overall Survival of CCA patients in study cohort.

|  | | Multivariate Analysis | | |
| --- | --- | --- | --- | --- |
| Factors | Univariate P | HR | 95% CI | P |
| Age (>60y vs ≤60y) | 0.249 | - | - | - |
| Gender (Male vs Female) | 0.832 | - | - | - |
| CA19-9 (>37 vs ≤37) | 0.480 | - | - | - |
| Histologic differentiation (P vs M/W) | 0.958 | - | - | - |
| TNM stage (III-IV vs I-II) | 0.042 | - | - | - |
| Lymph node invasion(pN1 vs pN0) | 0.039 | - | - | - |
| Akirin2(High vs Negative/Low) | 0.018 | 2.058 | 1.046-4.048 | 0.036 |
