# Supplementary material for: Akirin2 is modulated by miR-490-3p and facilitates angiogenesis in cholangiocarcinoma through the IL-6/STAT3/VEGFA signaling pathway
Source: Cell Death Dis. 2019 Mar 18;10(4):262. doi: 10.1038/s41419-019-1506-4 (PMC6423123; doi:10.1038/s41419-019-1506-4)
Supplement: Supplementary file 10 — Table S3 [file 41419_2019_1506_MOESM10_ESM.doc]

Table S3. Primer sequences and shRNA oligonucleotides information.

| Gene/miRNAs | Sequence |
| --- | --- |
| Akirin2 | Forward primer: 5′-TCTACGGCAGGTTGGGATGAT-3′  Reverse primer: 5′-AGCAGGCTGTTCTCCATATCG-3′ |
| GAPDH | Forward primer: 5′-GGGAGCCAAAAGGGTCAT-3′  Reverse primer: 5′-GAGTCCTTCCACGATACCAA-3′ |
| miR-130a-3p | Forward primer: 5′-CGCGCAGTGCAATGTTAAAA-3′  Reverse primer: 5′-AGTGCAGGGTCCGAGGTATT-3′ |
| miR-130b-3p | Forward primer: 5′- GCGCAGTGCAATGATGAAA-3′  Reverse primer: 5′-AGTGCAGGGTCCGAGGTATT-3′ |
| miR-139-5p | Forward primer: 5′-CGCGTCTACAGTGCACGTGTC-3′  Reverse primer: 5′-AGTGCAGGGTCCGAGGTATT-3′ |
| miR-140-5p | Forward primer: 5′-CGCGCAGTGGTTTTACCCTA-3′  Reverse primer: 5′-AGTGCAGGGTCCGAGGTATT-3′ |
| miR-142-3p | Forward primer: 5′-GCGCGTGTAGTGTTTCCTACTT-3′  Reverse primer: 5′-AGTGCAGGGTCCGAGGTATT-3′ |
| miR-301a-3p | Forward primer: 5′-CGCGCAGTGCAATAGTATTGT-3′  Reverse primer: 5′-AGTGCAGGGTCCGAGGTATT-3′ |
| miR-301b | Forward primer: 5′-CGCGCAGTGCAATGATATTGT-3′  Reverse primer: 5′-AGTGCAGGGTCCGAGGTATT-3′ |
| miR-454-3p | Forward primer: 5′-GCGCGTAGTGCAATATTGCTTA-3′  Reverse primer: 5′-AGTGCAGGGTCCGAGGTATT-3′ |
| miR-490-3p | Forward primer: 5′- CGCAACCTGGAGGACTCC-3′  Reverse primer: 5′-AGTGCAGGGTCCGAGGTATT-3′ |
| U6 | Forward primer: 5′-GCTTCGGCAGCACATATACTAAAAT-3′ |
| sh-Akirin2-1 | 5′-CAGCATCCTCACCATTAAA-3′ |
| sh-Akirin2-2 | 5′-TCAAATAATGCGACGATAT-3′ |
| sh-Akirin2-3 | 5′-CAGAACAAATTCTGTACAA-3′ |
| sh-NC | 5′-TTCTCCGAACGTGTCACGT-3′ |
