# Supplementary material for: Akirin2 is modulated by miR-490-3p and facilitates angiogenesis in cholangiocarcinoma through the IL-6/STAT3/VEGFA signaling pathway
Source: Cell Death Dis. 2019 Mar 18;10(4):262. doi: 10.1038/s41419-019-1506-4 (PMC6423123; doi:10.1038/s41419-019-1506-4)
Supplement: Supplementary file 11 — Supplementary figure legends [file 41419_2019_1506_MOESM11_ESM.doc]

**Supplementary figure legends**

**Fig. S1** Akirin2 mRNA levels were detected in 36 human CCA tissue specimens and 9 normal tissue samples from TCGA.

**Fig. S2** The knockdown efficiencies of two different shRNAs targeting Akirin2 are detected. **(A)** Infected cells were examined by phase contrast microscopy and fluorescent microscopy and high infection efficiency was seen in these cells under fluorescent microscopy. **(B)** Western blot analysis was conducted to evaluate the reliability of Akirin2 antibody and examine the efficiency of Akirin2 knockdown. Magnification, ×100 (A). Scale bar, 200 μm (A).

**Fig. S3** Overexpression of Akirin2 promotes **CCA** proliferation, migration and invasion *in vitro*. **(A)** Infected HuCCT1 cells were examined by phase contrast microscopy and fluorescent microscopy and high infection efficiency was seen in these cells. **(B)** Western blot analysis was employed to examine the efficiency of Akirin2 overexpression. **(C)** Proliferation curves were determined in Akirin2 stable overexpressing HuCCT1 cells by CCK-8 assays. **(D)** Colony-forming abilities were measured in Akirin2 stable overexpressing HuCCT1 cells by clonogenic assays. **(E)** Wound healing assay was performed to measure the migration ability of various cells as indicated. **(F)** Transwell assays were used to detect the migration and invasive capacities in Akirin2 stable overexpressing HuCCT1 cells. Magnification, ×40 (E), ×100 (A), ×200 (F). Scale bar, 100 μm (F), 200 μm (A), 500 μm (E). *, P < 0.05; **, P < 0.001. Data are shown as mean ± SD of at least three independent experiments.

**Fig. S4** Overexpression of Akirin2 promotes CCA tumor growth *in vivo*. **(A)** Xenograft tumors were generated by injecting HuCCT1 cells overexpressing Akirin2 or carrying a negative control vector. **(B)** The growth of xenograft tumors was measured by volume. **(C)** The tumor weight was recorded. **(D)** Ki-67 staining of the xenograft tumors is shown. Magnification, ×40 (D). Scale bar, 500 μm (A). *, P < 0.05.

**Fig. S5** E-cadherin, β-catenin, Vimentin and N-cadherin protein levels were detected in Akirin2 stable overexpressing HuCCT1 cells by western blotting.

**Fig. S6** Akirin2 overexpression promotes tumor angiogenesis. **(A)** The levels of VEGFA were detected in Akirin2 overexpressing cells and empty vector cell supernatants by ELISA assay. **(B)** Akirin2 promoted tumor-induced HUVEC migration according to Transwell migration assays**. (C-D)** Akirin2 promoted tumor-induced HUVEC angiogenesis according to tube formation assays and Aortic ring sprouting assay**. (E)** The levels of IL-6, STAT3, pSTAT3 and VEGF were detected in Akirin2 overexpressing cells and empty vector cells by Western blot. Magnification, ×40 (C), ×100 (D), ×200 (B). Scale bar, 500 μm (C), 200 μm (D), 100 μm (B). *, P < 0.05; **, P < 0.001. Data are shown as mean ± SD of at least three independent experiments.

**Fig. S7** Luciferase reporter assay was performed in HuCCT1 cells, with cotransfection of a luciferase construct fused with the wild-type or site mutant 3′-UTR of Akirin2 and pre-miR-490-3p or miR-NC. Luciferase activity is reported relative to that of Renilla.
